# Dr. Walker, on Mr. Goldson's Pamphlet

**Published:** 1805-02-01

**Authors:** John Walker

**Affiliations:** Salisbury Square


### *To the Editors of the Medical and Physical Journal.*

GENTLEMEN,

MORE 'Cases of small-pox subsequent to vaccination, by William Goldson,' have just been offered to the world. How is it that this gentleman still continues to keep groping in the dark, when he has himself been the cause of so much light breaking out in every quarter? even in that where he continues to bewilder himself in 'his attempt at reform in the practice of vaccination?' How is it that he yet remains so insensible to the immense importance of his subject, as to think of taking up his own and his reader's time in adjusting how far those who have written on the subject have been gentlemanly, &c.? He says, moreover, the simple egoist, "Those who know me can best appreciate the purity of my intentions."* While, however, he has laid hold of certain errors committed in and about town, and published them in support of his own, he has done himself the justice to abstain from the attempt to strengthen his cause from the publication mentioned in his last sheet. I have myself vainly attempted to

* A remark which he probably, happily, makes with all the consciousness of rectitude; but which he ought to have remembered is the common place language of the Charlatans, even of the notorious one whom he mentions in his last sheet, and who made unsuccesful efforts in 1798, to introduce into Paris, where I happened to be at the time, his *remedes pour l'indegestion et toutes sortes desmaux de l'estomac.*

to get it brought into discussion in several medical societies; but medical men every where seem to consider the pamphlet to originate in "Evil," and think it unworthy of notice.

W. G. says, in his Preface, 'from the result of experiments it was' (for—it was—read—I) 'concluded that inoculated cow-pox is only a temporary prevention.' And 'A few months only had elapsed before this conclusion was most decidedly confirmed in the practice of some the most eminent in the profession.'

I believe there is not any man of *character* in the profession, who would subscribe to W. G's opinion of vacciolation being only a temporary security; if there be such an one to be found, will he favour us with the address of his hardy compeer? 'Hence a spirit of inquiry has been excited.' The late Numbers in your Journal very satisfactorily exhibit many of the results of the experimental inquiries. They confirm the permanency of the protection of vacciola.

The author makes a great affair of inoculating on the hand instead of the arm, which, however, is a very common mode, especially among foreigners. All the matter used in the expedition, after its leaving Malta, was had from such inoculations. A military Surgeon (Mansen) had inoculated children in that way, on board the Diadem, on our going up, and on our arrival at Marmorice, and supplied me with these subjects. It sometimes happens that female relations, on my inoculating infants, mention to me that they themselves have never had the Small-pox; and, if I cannot prevail on them to let me vacciolate them in the usual way, I succeed in getting them to let me show them on the hand how trifling the puncture is. This produces more inflammation and pain than in the usual way; but they forgive me the 'pious fraud' I have exercised, on being assured that they are protected. Any difference in the appearance of the pocks produced in different parts of the body, does not appear to me to be worthy of any particular notice.

The Author, in his latter production, which he prefaces with *Omnia patefacienda, &c.* has been less careful in substantiating his statements than in the former; but in it the *invidia patefacta* is most strikingly prominent. The long pamphlet in question, of 150 pages, as far as I have read of it, reminds me of the Fable, *Parturiunt montes nascitur mus.*

*Salisbury Square*, 10, *j*. 1805. JOHN WALKER.

